# Long-Term Outcome of an HIV-Treatment Programme in Rural Africa: Viral Suppression despite Early Mortality

**DOI:** 10.1155/2011/434375

**Published:** 2010-11-21

**Authors:** Roos E. Barth, Hugo A. Tempelman, Robert Moraba, Andy I. M. Hoepelman

**Affiliations:** ^1^Department of Internal Medicine and Infectious Diseases, University Medical Centre Utrecht, F02.126, Postbus 85500, 3508 GA Utrecht, The Netherlands; ^2^Ndlovu Medical Centre, Elandsdoorn, P.O. Box 1508, Groblersdal 0470, South Africa

## Abstract

*Objective*. To define the long-term (2–4 years) clinical and virological outcome of an antiretroviral treatment (ART) programme in rural South Africa. *Methods*. We performed a retrospective observational cohort study, including 735 patients who initiated ART. Biannual monitoring, including HIV-RNA testing, was performed. Primary endpoint was patient retention; virological suppression (HIV-RNA < 50 copies/mL) and failure (HIV-RNA > 1000 copies/mL) were secondary endpoints. Moreover, possible predictors of treatment failure were analyzed. *Results*. 63% of patients (466/735) have a fully suppressed HIV-RNA, a median of three years after treatment initiation. Early mortality was high: 14% died within 3 months after treatment start. 16% of patients experienced virological failure, but only 4% was switched to second-line ART. Male gender and a low performance score were associated with treatment failure; immunological failure was a poor predictor of virological failure. *Conclusions*. An “all or nothing” phenomenon was observed in this rural South African ART programme: high early attrition, but good virological control in those remaining in care. Continued efforts are needed to enrol patients earlier. Furthermore, the observed viro-immunological dissociation emphasises the need to make HIV-RNA testing more widely available.

## 1. Introduction

Sub-Saharan Africa houses most of the 33 million people who are human immunodeficiency virus- (HIV-) infected globally. The scale-up of antiretroviral treatment (ART) roll-out that has taken place in this region over the last decade is impressive. Consequently, an increasing number of treatment-outcome data from African ART programmes has been published. In spite of frequently observed high early mortality rates, ART provides clear benefits for most HIV-infected patients. Typically, improvements in clinical, immunological, and virological outcome measurements are seen within months after starting treatment. However, long-term data (more than one year of followup) are less readily available [1, 2]. Moreover, most available data come from urbanized areas, and for many ART programmes regular HIV-RNA monitoring is not feasible. Clinical or immunological decline is frequently used to predict treatment failure in low-income countries (LICs). However, several studies portray limited correlation between such parameters and virological failure [3–5]. Furthermore, recently it has been shown that virological monitoring results in switching treatment earlier and at higher CD4 counts [6].

Ndlovu Medical Centre (NMC) is located in rural South Africa. Since the start of its ART programme, prospective virological monitoring has been done for all patients. Previously, we reported favourable short-term (one-year followup) results of patients receiving ART at NMC [7]. The objective of this report was to describe the long-term (2 to 4 years of followup) outcome. Due to consequent monitoring, longitudinal virological data could be analyzed. Furthermore, possible predictors for treatment failure were analyzed.

## 2. Methods

### 2.1. Cohort Description and Data Collection

Ndlovu MC is located in a poor, rural area of Limpopo, a province in the northeast of the Republic of South Africa. Details on NMC and its ART programme have been described elsewhere [7]. For the current study, all adults were included who started first-line ART at least 24 months before data-collection. A WHO clinical stage IV and a CD4+ T-cell count below 200 cells/mm^3^ were used as criteria for treatment eligibility. Treatment consisted of efavirenz or nevirapine plus stavudine or zidovudine and lamivudine. In case of virological failure, second-line ART was available. To this end, a boosted protease inhibitor (PI, lopinavir/ritonavir) was added to the nucleoside reverse transcriptase inhibitor (NRTI) backbone. 

Data were retrospectively collected from the medical charts. Body mass indices (BMI, weight/height^2^) are calculated at each visit. CD4+ T-cell counts (FACSCount system, Becton Dickinson Biosciences, San Jose, CA) and plasma HIV-RNA levels (system 340 bDNA analyzer, Bayer AG, Leverkusen, Germany, detection limit 50 copies/mL) are measured before treatment initiation and regularly thereafter (four times in the first and biannually during consecutive years). Patients are seen by HIV-counsellors during each clinic visit in order to provide information and to stimulate treatment adherence.

### 2.2. Outcome Definitions

Patient retention-rate was defined by the proportion of patients remaining in care at NMC, combined with the proportion of patients who were transferred to other clinics. Patient attrition was compiled of all-cause mortality and people who were lost to followup.

Achieving an HIV-RNA below 50 copies/mL was used to define virological suppression. An HIV-RNA over 1000 copies/mL after at least three months of ART was considered to be an indicative of virological failure. 

Patient attrition and virological failure were combined to define treatment failure. 

Immunological failure was defined according to the WHO guidelines as either (1) a CD4+ T-cell count after six months of therapy below 100 cells/mm^3^ or below the pre-therapy count or (2) a 50% decline from the on-treatment peak CD4+ T-cell count value [8].

### 2.3. Statistical Analysis

Patient retention-rate was the primary endpoint. Secondary outcomes were virological suppression and treatment failure. 

The negative and positive predictive values (NPV and PPV, resp.) of immunological failure, to determine virological failure, were calculated. Moreover, to define predictors of treatment failure, we compared the distribution of several determinants (Gender; Age; baseline BMI, CD4+ T-cell count, Karnofsky score and log HIV-RNA; (N)NRTIs used; employment status and years since treatment initiation) between patients with treatment success and those experiencing treatment failure. To this end, all patients who started ART were included in analyses. The same determinants were used to define predictors of virological failure. In order to minimize the influence of early mortality, only patients with more than three-month followup were included in these analyses. 

Continuous data were compared with the Student's *t*-test or the paired *t*-test as appropriate. Proportions were compared with the *χ* ^2^ test, and data that were not normally distributed were analyzed via the Mann-Whitney U or Wilcoxon test. Kaplan-Meier survival analysis was used to estimate the time from initiation of antiretroviral therapy to virological suppression and the time from initial suppression to subsequent virological failure. Odds ratios and 95% confidence intervals (95% CI) were calculated using logistic regression analysis. The Cox proportional-hazards model was used to identify independent predictors of the endpoints. To this end, determinants that were associated with the outcome in univariate analysis (defined as a *P*-value < .1) were included in multivariate analysis. A *P*-value ≤ .05 was considered statistically significant. Data were processed and statistical analyses were done using SPSS version 15.

## 3. Results

### 3.1. Patient Characteristics

The first 735 adults who started ART at NMC were included in analysis. Median duration between treatment initiation and data collection was 35 months (range 24–59). Women predominated (526/735, 72%). Unemployment rate was high at 70%; 24% of patients had less than six years school attendance. The median baseline CD4+ T-cell count (68 cells/mm^3^, IQR 20–140) and BMI (19.8 kg/m^2^, IQR 17.4–23.0) demonstrate that many patients were at an advanced stage of disease when initiating treatment. Most individuals initially received a stavudine-containing ART regimen (579/735, 79%); in 26% (151/579) of those patients, stavudine was subsequently switched to zidovudine, as they showed signs of neuropathy or lipodystrophy. Efavirenz was the most commonly prescribed NNRTI (58%. 426/735).

### 3.2. Clinical and Virological Outcome

**Figure 1:**
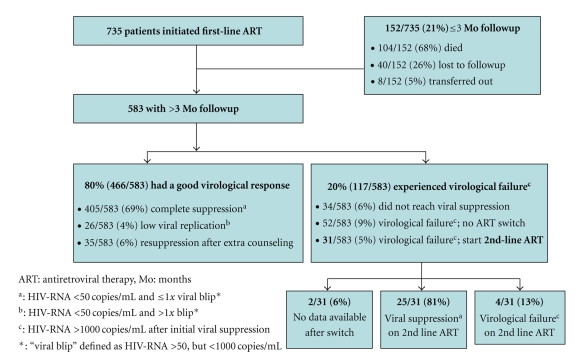
Virological treatment outcome.

Patient retention-rate at end of followup was 65% (476/735). Most (429/476, 90%) of these patients were still in care and on treatment at the NMC, but 10% (47/476) patients were transferred out to other clinics. The remaining 35% (259/735) of patients had either died or were lost to followup. Mortality was the main cause for attrition (171/259, 66%). Attrition typically occurred soon after treatment initiation; within three months, one out of five persons was not in care anymore (*n* = 152, Figure 1). 

562/735 patients (76%) achieved virological suppression on first line ART. Suppression was established at a median of 12 weeks after treatment initiation. Not achieving virological suppression was mainly due to early attrition (133/173, 77% within three months after treatment start). 

Longitudinal virological monitoring showed continued virological control on first-line ART in most patients (466/735, 63%). This proportion increased to 80% (466/583) if only those with more than 3-month followup were included. Virological failure was observed in 117/735 patients (16%: 20% {117/583} of those with >3 months followup). Median duration between initial response and subsequent failure was 18 months. Only 4% (31/735) of all patients who started first-line ART were switched to second-line ART. Most of them achieved virological suppression after switching (25/31, 81%). Virological outcome data are summarized in Figure 1.

**Figure 2:**
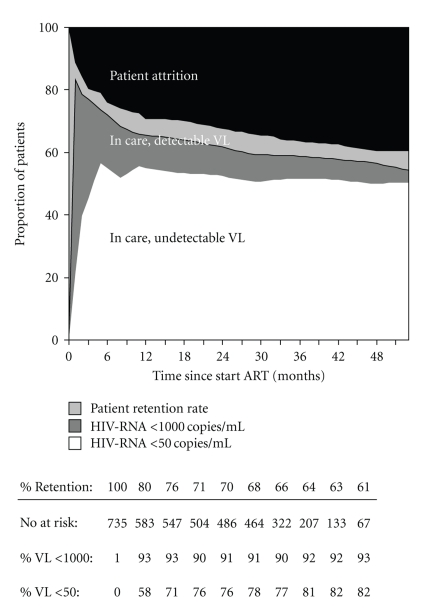
Viral replication during 1st line ART.

Ongoing viral replication was limited in patients receiving first-line ART. During complete followup, less than 10% of patients who remained in care had an HIV-RNA > 1000 copies/mL and the vast majority of patients (71–82%) showed complete, virological suppression (HIV-RNA < 50 copies/mL, Figure 2).

### 3.3. Predictors of Failure

Ninety-five patients showed evidence of immunological failure (13%). On the basis of immunological parameters, virological failure would have been missed in 66/117 patients (56%) and 44/95 patients (46%) would mistakenly have been classified as experiencing virological failure. Immunological failure therefore showed a PPV and NPV for virological failure of 54% and 87%, respectively. 

Overall, treatment failure (attrition and/or virological failure) occurred in 337 patients (46%) during followup. Of all baseline factors that were associated with treatment failure in univariate analysis (low BMI and CD4+ T-cell count, Karnofsky score ≤ 50, Zidovudine-use and gender), only a low performance (Karnofsky) score (OR 3.6, 95% CI 1.8–7.1) and male gender (OR 1.7, 95% CI 1.2–2.3) remained independently associated with the outcome after multivariate analysis (Table 1).

In patients with more than three-month followup, baseline CD4+ T-cell count (*P* = .03) and age (*P* = .07) were associated with virological failure in univariate analysis. Neither remained independently associated with this outcome after multivariate analysis.

## 4. Discussion

A median of three years after treatment initiation, half of the patients receiving ART in this rural South African clinic is still in care and has a fully suppressed HIV-RNA. Nearly a quarter of patients died, mostly early on. Sixteen percent of patients showed virological failure; only few of them were switched to second-line treatment. Apart from a low Karnofsky score at baseline, male gender was predictive of treatment failure. The correlation between immunological and virological failures on the other hand was minimal.

The high early mortality rate is probably largely attributable to the advanced clinical stage patients are in when seeking care, As has been described for other African cohorts, patients typically are in an advanced stage of their disease when seeking care [1, 9]. This probably explains the large number of people who died soon after starting ART. Regression analysis indeed shows a low performance score to be associated with treatment failure. Thus, continued efforts are needed to enrol patients earlier, before clinical illness becomes evident. Male gender also remained as an independent predictor of treatment failure in our analysis. Similar associations have been observed previously [10, 11], but others did not find gender to be of influence on ART outcome [12, 13]. In contrast, the CD4+ T-cell count did not remain significantly associated with treatment failure, suggesting that starting ART prior to clinical illness is of greater importance than merely avoiding a low CD4+ T-cell count. 

Drug-resistance development is a major concern when treating HIV. The virus can select drug-resistance mutations in the presence of suboptimal drug levels. In case of continued ART use in spite of ongoing viral replication, accumulation of resistance can occur [14–16]. Mortality was the main cause of treatment failure in our cohort. In addition, continued virological control was observed in the majority of patients who did remain in care. Due to this observed “all or nothing” phenomenon, the number of people experiencing ongoing viral replication in the presence of ART was limited. 

Regular HIV-RNA testing is not feasible in many LICs. In accordance with WHO-guidelines, treatment changes are frequently based on immunological criteria alone [8, 17]. Immunological failure was a poor predictor for experiencing virological failure in our cohort. Basing treatment decisions on immunological parameters would lead to unnecessary treatment switches in a substantial number of patients, as has been described previously [4, 5]. We however show, that the proportion of patients that would mistakenly continue a failing regimen if treatment decisions were merely based on immunological parameters is even larger. Such viro-immunological dissociation highlights the need for virological monitoring for all patients receiving ART. 

In the current study, regular (bi-annual) virological monitoring was performed. Still, a substantial number of patients continued their first-line ART after showing the first evidence of virological failure. The delay between virological failure and treatment switch is probably due to care-givers focusing on extra adherence counselling, prior to switching to the only available second-line regimen. On the basis of virological failure alone, 16% of patients in our cohort had an indication for a treatment switch. A recent review reported nearly twice as many patients in low and middle income countries needing a second-line regimen (26–32%, [17]), but others reported similar numbers to those observed in our cohort [18–20]. As most patients on second-line ART achieved virological suppression again, the number of people with ongoing viral replication may be reduced even further if treatment switches are made earlier. However, future cost-benefit analyses will have to determine the optimal time to switch. 

There are some limitations to our study. Diagnostics for underlying illnesses and documentation of adverse events were limited. Causes of attrition were therefore generally unknown. Moreover, due to the retrospective nature of our study, bias due to unmeasured determinants cannot be ruled out. 

In conclusion, in this rural South African ART programme with access to virological monitoring, early attrition was the main cause for treatment failure. Virological failure was limited in those remaining in care. Continued efforts are needed to enrol patients earlier into care. Furthermore, the observed viro-immunological dissociation emphasises the need to make HIV-RNA testing more widely available.

## Figures and Tables

**Table 1:** Predictors of treatment failure.

Determinant	Treatment Success* *n* = 398	Treatment Failure** *n* = 337	Univariate (*P*-value)	Multivariate (*P*-value)
Male gender *n* (%)	95 (24)	114 (34)	<.01	.01
Mean Age (years)	35.3	35.3	1.0	—
Mean BMI (kg/m_2_)	21.1	19.9	<.01	.4
Karnofsky score ≤50 *n* (%)	12 (3)	35 (11)	<.01	<.01
Median CD4 (cells/mm_3_)	84	54	<.01	.2
Mean 10log HIV-RNA	4.9	4.9	.8	—
Time since start ART *n* (%)			.3	—
(i) 2-3 years	217 (55)	162 (48)		
(ii) 3-4 years	121 (30)	111 (33)		
(iii) >4 years	60 (15)	60 (18)		
Efavirenz use *n* (%)	223 (56)	204 (61)	.2	—
Stavudine use *n* (%)	324 (82)	253 (76)	.05	.3
Unemployed *n* (%)	275 (74)	237 (79)	.1	—

Treatment success* patients who remained in care during followup and showed a good, virological response. Treatment failure**: a combined endpoint of patient attrition and virological failure. *n*: number of patients. %: percentage of patients. BMI: body-mass index. CD4: CD4+ T-cell count. ART: antiretroviral treatment.
